# Raman scattering and anomalous Stokes–anti-Stokes ratio in MoTe_2_ atomic layers

**DOI:** 10.1038/srep28024

**Published:** 2016-06-21

**Authors:** Thomas Goldstein, Shao-Yu Chen, Jiayue Tong, Di Xiao, Ashwin Ramasubramaniam, Jun Yan

**Affiliations:** 1 Department of Physics, University of Massachusetts, Amherst, Massachusetts 01003, USA; 2 Department of Physics, Carnegie Mellon University, Pittsburgh, PA 15213, USA; 3 Department of Mechanical & Industrial Engineering, University of Massachusetts, Amherst, Massachusetts 01003, USA

## Abstract

Stokes and anti-Stokes Raman scattering are performed on atomic layers of hexagonal molybdenum ditelluride (MoTe_2_), a prototypical transition metal dichalcogenide (TMDC) semiconductor. The data reveal all six types of zone center optical phonons, along with their corresponding Davydov splittings, which have been challenging to see in other TMDCs. We discover that the anti-Stokes Raman intensity of the low energy layer-breathing mode becomes more intense than the Stokes peak under certain experimental conditions, and find the effect to be tunable by excitation frequency and number of atomic layers. These observations are interpreted as a result of resonance effects arising from the C excitons in the vicinity of the Brillouin zone center in the photon-electron-phonon interaction process.

The coupling between photons, electrons, and phonons is central to understanding fundamental properties of condensed matter systems. In layered transition metal dichalcogenide (TMDC) semiconductors, electron-photon coupling enables investigation of the under-screened strong Coulomb interaction in reduced dimensions^1^ as well as the manipulation of spins and pseudo-spins^2^,^3^. The interaction of chiral phonons with electrons and circularly polarized photons is linked to pseudo-spin flip, and may lead to a novel valley phonon Hall effect, as predicted by a recent theory^4^. Despite much recent experimental progress^2^,^5^, the coupled photon-electron-phonon system in TMDCs is still not fully understood, and continues to be a focus of two dimensional (2D) materials research.

In this work we study such interaction effects in molybdenum ditelluride (MoTe_2_), a prototypical TMDC semiconductor, which has recently attracted a great deal of interest. Investigations of multi- and mono-layer MoTe_2_ transistor devices^6^,^7^,^8^ have probed the semiconductor’s band gap and transport properties, revealing ambipolar charge conduction channels, and a stable metallic T’ phase which is potentially a type-II Weyl semimetal^9^,^10^, and can be used to make Ohmic homojunction contacts^6^. Optically, monolayer MoTe_2_ features strong photoluminescence similar to other TMDCs, but it has by far the smallest optical bandgap at the K points, lying in the infrared around 1 eV^11^,^12^. As a result, the C excitons located near the Brillouin zone center (Γ)^13^ are in the visible range around 2.5 eV^11^, which we take advantage of to investigate the resonance effects reported here.

We use Raman Spectroscopy to probe the photon-electron-phonon coupling in MoTe_2_, revealing hard to access Raman modes and mode splitting in detail. We also observe a peculiar phenomenon in which the anti-Stokes Raman peak becomes more intense than the Stokes peak, which is contrary to the usual intuition from Boson statistics, and could potentially serve as a laser cooling channel for the atomically thin 2D crystals. Our further investigation into how the photon-electron-phonon coupling affects the anti-Stokes intensity reveals that the phenomenon is tunable according to laser wavelength and MoTe_2_ layer number. These experimental observations highlight the role of the C excitons, and provide insights into the lattice dynamics and electronic structures of molybdenum ditelluride.

## Results and Discussion

**Figure 1:**
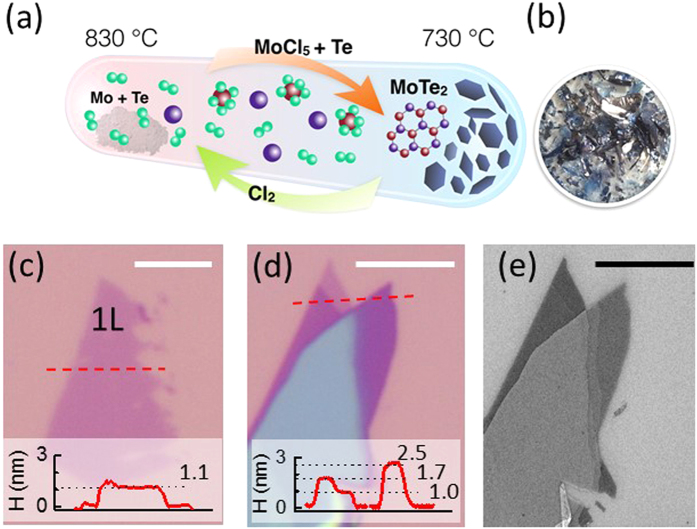
(**a**) Schematic illustration of the chemical vapor transport crystal growth process. (**b**) Image of grown MoTe_2_ crystals. (**c,d**) Optical microscope image and AFM trace [along the red dashed lines)] of mechanically exfoliated MoTe_2_ flakes. (**e**) SEM image of (**d**). All the scale bars are 5 μm.

The MoTe_2_ atomic layers used in this work are exfoliated from bulk crystals grown via chemical vapor transport with chlorine as the transport agent, as illustrated in Fig. 1a (more details in Methods). Typical sizes of the crystals (Fig. 1b) range from a few mm to 1 cm. Atomic layers of MoTe_2_ are exfoliated with Scotch tape and deposited on silicon with a 300 nm oxide layer or on fused silica substrates. Figure 1c,d show optical microscope images of typical atomic flakes of MoTe_2_.

We use several different methods to determine the atomic layer number. The atomic force microscope (AFM) characterization in Fig. 1c,d shows that the step height from substrate to monolayer in these two samples is 1.0 nm and 1.1 nm respectively, and that the step height from monolayer to bilayer and from bilayer to trilayer is about 0.7 nm, consistent with previous measurements^11^. The larger step height between monolayer and SiO_2_ substrate than between adjacent layers is frequently seen in AFM characterization. Supplementary Fig. S1 shows data on 10 monolayer samples; the AFM step height varies significantly from one sample to another, including five samples with step heights close to or above 1.4 nm, giving those samples an ambiguous layer number by AFM alone. However, despite having different step heights between the substrate and monolayer, optical contrast remains the same across different crystals with the same layer number. We confirmed the monolayer nature of those samples using Raman spectroscopy (details below), and combined this with the 0.7 nm interlayer step height to quantify multi-layer samples. We also performed further characterization with scanning electron microscopic (SEM) imaging of the MoTe_2_ flakes, as shown in Fig. 1e (taken at 5 KV). The mono-, bi-, and tri-layers are distinguishable under SEM, with the image becoming brighter with an increase in layer number, indicating that more secondary electrons are emitted from thicker samples.

**Figure 2:**
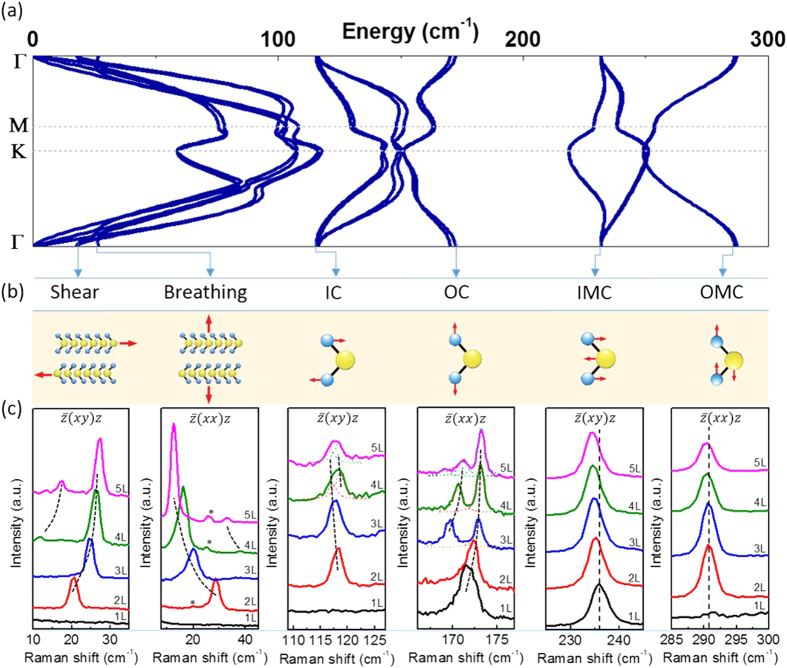
(**a**) Phonon dispersion of bilayer MoTe_2_ calculated with density functional theory (DFT). (**b**) Atomic displacements of the six prototypical TMDC Γ point optical phonons. The arrows between (**a,b**) connect the calculated Γ point optical phonons with the names we use to identify the lattice vibrations. (**c**) Experimental Raman spectra for all six phonon branches in 1L to 5L MoTe_2_; the spectra have been vertically shifted for clarity. The black dashed curves are guides to the eye. The small peaks in the 2^nd^ panel (*) are the shear mode peaks shown in more detail in the 1^st^ panel.

Atomic layers of hexagonal TMDCs host six prototypical zone-center optical phonons, shown in the bilayer dispersion (see Supporting Information for details of DFT calculation) and atomic displacement drawings of Fig. 2a,b, respectively. These include the shear mode (S), the breathing mode (B), the in-plane chalcogen vibration (IC), the out-of-plane chalcogen vibration (OC), the in-plane metal-chalcogen vibration (IMC), and the out-of-plane metal-chalcogen vibration (OMC)^14^. We measure the in-plane S, IC and IMC modes with cross polarization 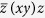 and the out-of-plane B, OC and OMC modes in parallel polarization 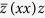 (see Methods for details of Raman set-up). Figure 2c shows the Raman spectra excited with the 532 nm (2.33 eV) laser light. The mode energies, in cm^−1^ (1 meV ≈ 8.06 cm^−1^), are given in Table 1; for the bilayer, values derived from DFT are given in parenthesis for comparison.

The occurrence and splitting of the observed Raman peaks can be compared with group theoretical predictions: Table 2 lists the symmetries and expected number of modes for the six types of zone-center optical phonons in one to five layers and in the bulk as derived from ref. 14. Except for the bulk B and OMC modes of B_2g_ symmetry^15^, all modes that are even under inversion/mirror-reflection operation (*i*/σ_h_) are Raman active (bold text in Table 2). For 1L-MoTe_2_ the S and B modes are absent since interlayer vibrations only occur in multi-layers, and the odd IC and OMC modes are not Raman active; this is in agreement with the observation of only the OC and IMC modes in the monolayer (black) spectra in Fig. 2c. Bilayer MoTe_2_ has one Raman active mode for each of the six types of zone center optical phonons, consistent with the six peaks observed in bilayer (red) spectra.

For samples thicker than bilayer the Raman spectra display Davydov splitting due to interlayer interactions. We observe two B and two S modes in 4L and 5L samples; two OC modes in 3L and 4L samples; and three OC modes in 5L samples, in perfect agreement with the theoretical expectations in Table 2. The small additional peaks present in Fig. 2c 2^nd^ panel are the same breathing mode peaks as in the 1^st^ panel; they are present under both the z(xy)z and z(xx)z configuration, though their intensity is much less than that of the breathing mode, which is only present under the z(xx)z configuration. The IC mode appears to display non-monotonic dependence on layer numbers, and develops an asymmetric shape for 4L and 5L samples. This indicates that the IC spectra in 4L and 5L samples are composed of two peaks as suggested by the theory in Table 2. With this interpretation (dashed fitting curves of Fig. 2c), the IC vibrations are composed of two sets of modes consistently red-shifting with increasing number of layers. The IMC is expected to split in a manner similar to OC, and OMC similar to IC, but neither were observed experimentally: IMC and OMC display a single symmetric peak in multi-layer MoTe_2_ in Fig. 2c. This might be due to either the splitting being small compared with the linewidth or the split modes having too small Raman cross-section. We note that the former interpretation is in agreement with the theoretical phonon dispersion calculation in Fig. 2a where the even (A_1g_) and odd (A_2u_) OC vibrations in 2L-MoTe_2_ have the largest Davydov splitting.

In the other four hexagonal TMDCs, (Mo,W)(S,Se)_2_, the OC and IMC modes are the dominant Raman features, and the B and S modes become observable if low-energy stray light is cut, but the IC and OMC modes tend to evade most Raman scattering measurements^14^,^16^,^17^,^18^,^19^,^20^,^21^,^22^ unless less-common ultraviolet lasers are used^17^. However, because of MoTe_2_’s small optical band gap^11^, the visible lasers used here can access the C excitons arising from electron states in the vicinity of the Γ point^23^. Recent theoretical calculations^13^ show that the C exciton is composed of six nearly degenerate states involving the topmost valence band and the three lowest conduction bands, in contrast to the A and B excitons where only one conduction and one valence band near the K points are involved. The availability of multiple bands and states provides more electronic degrees of freedom for electron-phonon interaction, which in turn assist the excitation of all of the zone-center optical phonons in MoTe_2_. In capturing all six zone center modes, our Raman spectra in Fig. 2c and Table 1 constitute a comprehensive data set for TMDC lattice dynamics, in agreement with other recent works on MoTe_2_^24^,^25^.

**Figure 3:**
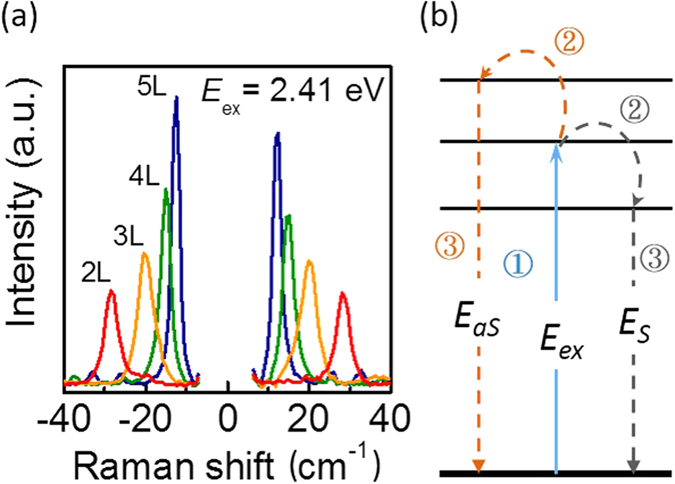
(**a**) Experimental Raman data of MoTe_2_ under a 2.41 eV excitation, showing the evolution of the breathing mode peak position and intensity with layer number. The anti-Stokes intensity is anomalously greater than the Stokes intensity. (**b**) Illustration of the zone-center optical-phonon Raman scattering process.

**Figure 4:**
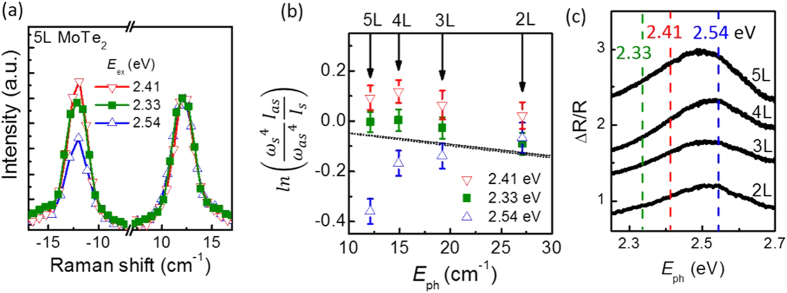
(**a**) Experimental Raman data for the breathing mode of 5 layer MoTe_2_ using 2.33, 2.41, and 2.54 eV excitations. Stokes intensities have been normalized, showing the difference in anti-Stokes intensity between different excitation energies. (**b**) 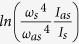 as a function phonon mode energy in cm^−1^. Each breathing mode energy corresponds to a different layer number: 5 layer to 2 layer, from low to high energy. The grey band shows the expected value if there were no resonance effects, with the bounding black dotted lines determined by the experimental temperature uncertainty in our measurements (see Supporting Information). (**c**) Differential reflectance data for 2 layer to 5 layer MoTe_2_ in the energy region of the C exciton. Dashed lines indicate the energy of the laser excitations used for Raman measurements in panels (**a,b**).

In addition to enabling observation of all six branches of zone center lattice vibrations, the C excitons in the photon-electron-phonon interaction Raman process have interesting impacts on Raman intensity. In particular we have found that the resonances have dramatic effects on the Stokes and anti-Stokes Raman signatures of the low energy breathing modes. Figure 3a shows the breathing mode Stokes and anti-Stokes peaks for 2 through 5 layers, using the 2.41 eV laser excitation. (The Raman intensities in Figs 3 and 4 are calibrated; see Supporting Information.) As expected, the anti-Stokes peaks are distributed symmetrically in Raman shift about zero with respect to the Stokes peaks. However we also observe that the anti-Stokes peaks are more intense than the Stokes peaks, an unusual occurrence not present in most systems.

In a typical, lowest-order Raman scattering process for zone-center optical phonons, there are three steps involved as shown in Fig. 3b:

1. The electronic system absorbs a photon and creates an electron-hole pair;
2. The electron-hole pair creates (Stokes) or absorbs (anti-Stokes) a phonon;
3. The electron-hole pair recombines and emits the scattered photon.

The first and the third steps are linked to electron-photon interaction while the second step is determined by electron-phonon interaction. The rate of step 2 is proportional to the phonon creation or destruction operator acting on the system squared, giving a factor of (*n* + *1*) or *n*, respectively, where *n* is the phonon occupation number. As phonons are bosonic excitations with zero chemical potential, 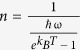 where 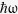 is the phonon energy and 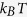 is the thermal energy. For step 3, the recombining electron-hole pair acts similarly to a radiating dipole, whose emission rate carries a factor of 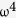 according to classical field theory^26^. Thus, the anti-Stokes to Stokes intensity ratio is predicted to be


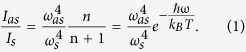


Note that the impact of the polynomial 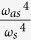 is typically much smaller than the exponential 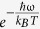 thus *I*_*as*_ is expected to be less intense than *I*_*s*_, a condition which is ubiquitously observed in most material systems.

The anomalously intense anti-Stokes peaks suggest that some other factor not included in Equation (1) plays an important role in determining the Raman cross section. Step 1 in Fig. 3b is shared by the Stokes and anti-Stokes process, and the differences of step 2 are purely in occupation number, so neither can explain the anti-Stokes intensity. This leaves elements of step 3 not included in the classical theory of radiating dipoles; in particular any resonances between the final emitted Stokes and anti-Stokes photons and the electronic structure of the crystal. For our frequency ranges this is, as mentioned above, the C exciton, which will have a different resonance effect on the Stokes and anti-Stokes emitted photons due to their different energies.

The resonance effect becomes even clearer when we systematically measure the calibrated breathing mode intensities using different laser lines. Figure 4a shows Raman data (normalized by Stokes intensities) for 5L-MoTe_2_ excited by 488 nm (2.54 eV), 514 nm (2.41 eV), and 532 nm (2.33 eV) respectively. The anti-Stokes to Stokes intensity ratio depends sensitively on the incident laser excitation: at 2.33 eV, the anti-Stokes peak has about the same intensity as the Stokes peak; at 2.41 eV and 2.54 eV, the anti-Stokes intensity is above and below the Stokes, respectively. From these observations, we introduce a resonance correction factor, *R*, into Equation (1), such that


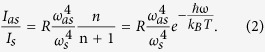


In Fig. 4b we plot 
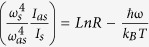
 as a function of the breathing mode phonon energy for 2 through 5 layer MoTe_2_ with all three excitation wavelengths. In the absence of any correction, i.e. *R* = 1 in Equation (2), the experimental data are expected to fall within the gray band, where the two bounding dotted black lines specify the temperature uncertainty during our experiment (see Supporting Information). The experimental data show significant deviation from *R* = 1, with the enhancement and suppression of the anti-Stokes to Stokes ratio mostly consistent across layer number.

To further establish the connection between the C exciton and the laser-wavelength/atomic-layer-number dependent *R* factor, we collected differential reflection data, presented in Fig. 4c, showing the C exciton resonance for 2 through 5 layer MoTe_2_ (see Methods). The data strongly imply that the observed deviations of the *R* factor from unity are correlated with the overall shape and evolution of the C exciton: the measured 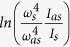 for 5L samples has the largest variations at different laser excitations, consistent with the fact that its exciton resonance is the most sharply peaked. For 3L to 5L samples, 2.54 eV lies on the opposite side of the exciton peak from 2.41 and 2.33 eV; this corresponds to the swapping from resonance suppression to resonance enhancement of the anti-Stokes intensity. The slopes of the differential reflection for 2.33 and 2.41 eV are roughly constant; this corresponds to the roughly constant *R* value across different numbers of layers for these energies. In contrast the slopes for 2.54 eV excitations have significant variations, as do the resonance effects in different numbers of layers.

We finally remark that while other materials with exciton effects may also exhibit an anomalous Stokes–anti-Stoke ratio, it is quite rare to have the anti-Stokes peak to actually be more intense than the Stokes peak. Also we take note that the Stokes–anti-Stokes photon energy difference for the 5L-MoTe_2_ breathing mode is only about 3 meV, corresponding to about 0.15% of the total photon energy. It is quite remarkable that the C exciton resonance at room temperature can amplify this minute change in energy to switch the relative Stokes–anti-Stokes Raman cross-section. The reversed intensity ratio in fact indicates that more phonons are removed from, rather than created in, the crystal, potentially forming a laser-cooling channel in the system. Indeed, the observation of intense anti-Stokes in another semiconductor system (CdS nanoribbons) is accompanied with strong laser cooling effects^27^. We thus surmise that our observations point to the uncharted field of laser cooling of atomically thin 2D crystals, corroborating a recent study of luminescence upconversion in WSe_2_^28^.

## Conclusion

We have exfoliated few layer MoTe_2_ from chemical vapor transport grown crystals. Using polarization-resolved Raman scattering we have unambiguously identified all six types of zone-center optical phonons. Splitting of these phonons, where observed, has the correct theoretically predicted number of branches. We discovered that the anti-Stokes intensities deviate from their expected values, to the point of being more intense than the Stokes intensities, with the intensity ratio tunable via resonance with the C exciton. A stronger anti-Stokes peak implies that more phonons are annihilated than created in the crystal suggesting that, from the electron-photon-phonon interaction process, anti-Stokes Raman scattering could provide a cooling channel in atomically thin TMDC crystals.

## Methods

### Bulk MoTe_2_ crystal growth

We grew bulk MoTe_2_ crystals using chemical vapor transport with chlorine as the transport agent, as illustrated in Fig. 1a. A horizontal three-zone Carbolite furnace provides a high temperature reaction zone and a low temperature growth zone. Following the work of Ubaldini *et al*.^29^, Mo, Te, and MoCl_5_ powders are placed in a fused silica tube, 18 mm in diameter and 300 mm in length. The purity of the source materials are Mo 99.9%, Te 99.997%, and MoCl_5_ 95% (Sigma Aldrich). Total Mo and Te are kept in a stoichiometric 2:1 ratio with sufficient MoCl_5_ to achieve a Cl density of 2 mg/cm^3^. The tube is pump-purged with ultra-high purity argon gas and sealed at low pressure prior to growth. The reaction and growth zones were kept at 830 °C and 730 °C respectively for 100 hours. During cooling the temperature profile is inverted with the growth zone being roughly 100 °C hotter than the reaction zone so that chloride contaminants preferentially precipitate in the hot zone, away from the MoTe_2_ crystals.

### Raman Scattering and Differential Reflection

We collect Raman data using linearly-polarized light from an Argon laser or a frequency doubled Nd:YAG solid state laser to excite the samples in a back scattering geometry. The scattered light is collected with a 100x objective lens, dispersed by a triple stage spectrometer, and detected by a liquid nitrogen cooled CCD. In the collection path, a broadband polarization rotator and a linear polarizer are used to selectively detect scattered light with polarization either parallel 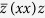 or perpendicular 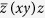 to that of the incident light. With a combination of a Bragg diffraction grating (OptiGrate) and a Horiba T64000 triple stage spectrometer operating in the subtractive mode, we were able to observe low energy phonon modes down to less than 10 cm^−1^. Data for 488 and 514 nm excitations were taken at 100 μW for 120 s; data for 514 nm excitations were taken at 70 μW for 120 s. The heating effects of these powers were accounted in Fig. 4 (see Supporting Information).

We obtain differential reflection data by exfoliating MoTe_2_ atomic layers onto fused silica. We then shine a white laser (NKT Photonics) on the samples and collect the reflection spectrum, which is subtracted from and normalized by the substrate reflection.

## Additional Information

**How to cite this article**: Goldstein, T. *et al*. Raman scattering and anomalous Stokes–anti-Stokes ratio in MoTe_2_ atomic layers. *Sci. Rep.* **6**, 28024; doi: 10.1038/srep28024 (2016).

## Supplementary Material

Supplementary Information

## Figures and Tables

**Table 1:** Energy (cm^−1^) of the observed zone-center optical phonon modes from 1L to 5L MoTe_2_.

Mode	1L	2L	3L	4L	5L
S		19.6 (17.9)	24	10.4/25.3	16.7/26.2
B		28.6 (26.5)	20.9	16.2/37.9	12.6/32.9
IC		118.1 (116.5)	117.6	118.1	117.8
OC	171.5	172.4 (172.3)	172.9/169.6	173.1/170.5	173.2/171.1/169.0
IMC	236.0	235.2 (231.7)	234.9	234.7	234.5
OMC		290.7 (287.2)	290.6	290.4	290.3

For the bilayer, DFT results are included in the parentheses.

**Table 2:** Symmetry representation for phonon modes in bulk and few layer TMDCs^14^.

# of Layers	Sym. Grp.	σ_h_/i Sym.	S	B	IC	OC	IMC	OMC
1	D_3h_	+	−	−	−	**1 A’_1_**	**1 E’**	−
		−	−	−	1 E”	−	−	1 A”_2_
2	D_3d_	+	**1 E_g_**	**1 A_1g_**	**1 E_g_**	**1 A_1g_**	**1 E_g_**	**1 A_1g_**
		−	−	−	1 E_u_	1 A_2u_	1 E_u_	1 A_2u_
3	D_3h_	+	**1 E’**	**1 A’_1_**	**1 E’**	**2 A’_1_**	**2 E’**	**1 A’_1_**
		−	1 E”	1 A”_2_	2 E”	1 A”_2_	1 E”	2 A”_2_
4	D_3d_	+	**2 E_g_**	**2 A_1g_**	**2 E_g_**	**2 A_1g_**	**2 E_g_**	**2 A_1g_**
		−	1 E_u_	1 A_2u_	2E_u_	2 A_2u_	1 E_u_	2 A_2u_
5	D_3h_	+	**2 E’**	**2 A’_1_**	**2 E’**	**3 A’_1_**	**3 E’**	**2 A’_1_**
		−	2 E”	2 A”_2_	3 E”	2 A”_2_	2 E”	3 A”_2_
bulk	D^4^_6h_	+	**1 E_2g_**	**1 B_2g_**	**1 E_1g_**	**1 A_1g_**	**1 E_2g_**	**1 B_2g_**
		−	−	−	1 E_2u_	1 B_1u_	1 E_1u_	1 A_2u_

The third column specifies whether the mode is even or odd under horizontal mirror plane reflection/inversion. Raman active modes (in back scattering geometry) are colored in bold red.
