# Supplementary material for: Raman scattering and anomalous Stokes–anti-Stokes ratio in MoTe2 atomic layers
Source: Sci Rep. 2016 Jun 21;6:28024. doi: 10.1038/srep28024 (PMC4914966; doi:10.1038/srep28024)
Supplement: Supplementary Information [file srep28024-s1.pdf]

Supplementary Information

Raman scattering and anomalous Stokes–anti-Stokes ratio in $MoTe_2$ atomic layers

Thomas Goldstein[1†], Shao-Yu Chen[1†], Jiayue Tong[1], Di Xiao[2], Ashwin Ramasubramaniam[3]

and Jun Yan[1]*

[1]Department of Physics, University of Massachusetts, Amherst, MA 01003, USA

[2]Department of Physics, Carnegie Mellon University, Pittsburgh, PA 15213, USA

[3]Department of Mechanical & Industrial Engineering, University of Massachusetts, Amherst, MA 01003, USA

[†]These authors contributed equally to this work.

[*]Corresponding Author: Jun Yan. Tel: (413)545-0853 Fax: (413)545-1691

E-mail: yan@physics.umass.edu

S1. Thickness characterization by Atomic Force Microscopy

We measured the thickness of 1L- $MoTe_2$ by Atomic Force Microscopy (AFM). Figure S1a shows the optical microscope image of 10 samples with monolayer $MoTe_2$ flakes. We preformed AFM measurements around the 1L and extracted the step height from substrate to monolayer. The traces along the dash lines are measured and shown in the Figure S1b. We notice that the step height of monolayer $MoTe_2$ exhibits large variations, ranging from 0.9 to 2.4 nm. As discussed in main text, it might be due to the surface contamination or atomic water layer which is encapsulated between the flakes and the substrate. We further summarized the step height for 10 samples with the bar chart in Figure S1c.

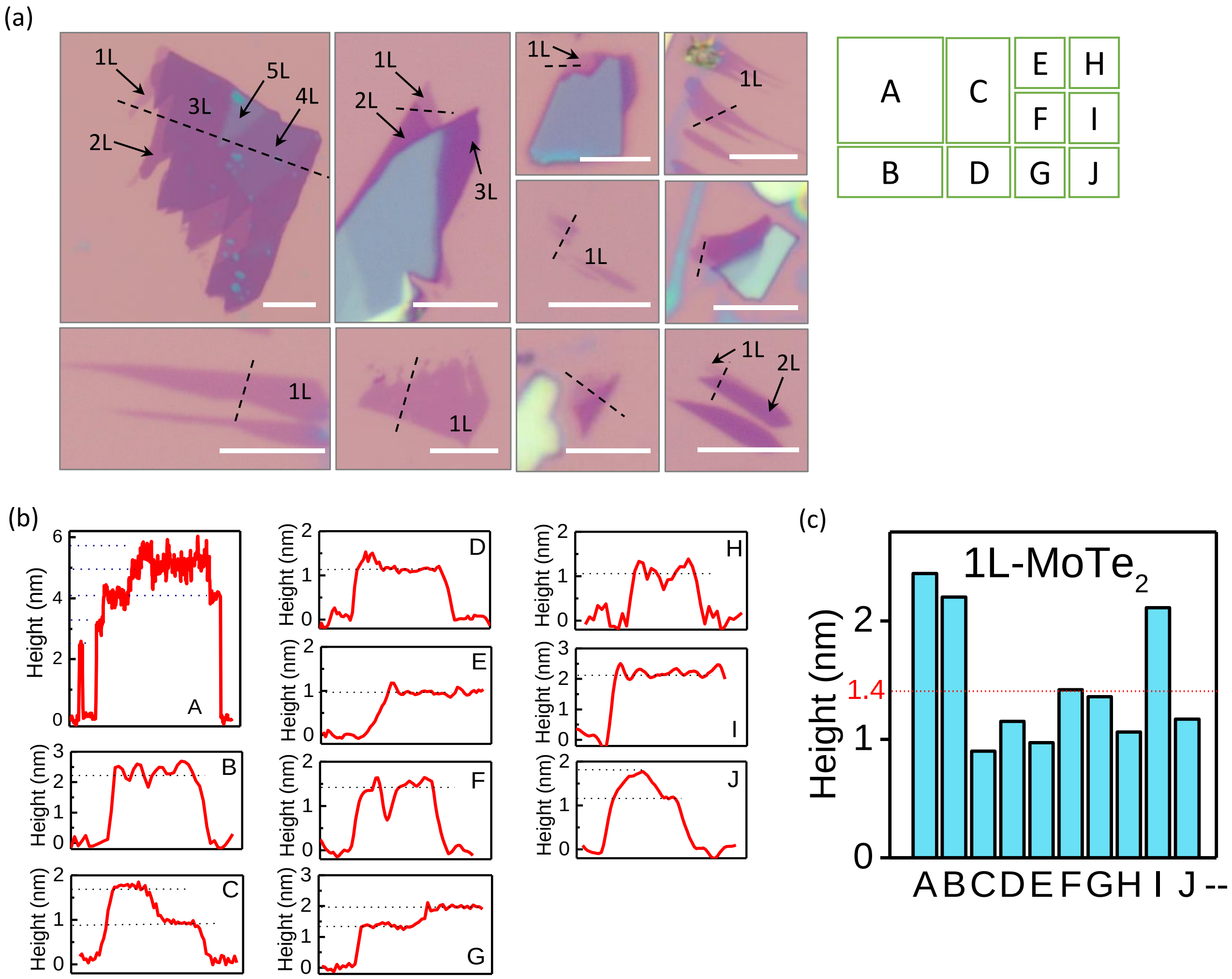

Figure S1. (a) The optical microscope images of 10 different $MoTe_2$ samples on Si/SiO_2 substrate, from A to J. All the scale bars are 5 μm. (b) Traces of monolayer step height are measured for sample A to J, along the dash lines shown in (a). (c) The bar chart shows the height of monolayer $MoTe_2$ that varies from 0.9 nm to 2.4 nm. The red dash line indicates the thickness of 1.4 nm, which corresponds to the expected height of two atomic layers of $MoTe_2$.

S2. Phonon dispersion calculation.

The $MoTe_2$ bilayer was modeled using density functional theory (DFT) as implemented in the Vienna Ab Initio Simulation Package (VASP) [1]. The projector-augmented wave (PAW) method[2,3] was employed to represent core and valence electrons. The 4p, 5s, and 4d electrons of Mo, and the 5s and 5p electrons of Te were treated as valence. From convergence tests, a plane-wave cutoff of 400 eV was chosen with a Brillouin zone sampling equivalent to a Γ-centered 8×8×1 mesh for the $MoTe_2$ primitive cell. For relaxation of the primitive cell, electronic wavefunctions were converged to within 10^{-4} eV in conjunction with a Gaussian smearing 0.05 eV. In-plane cell vectors and atomic positions for the $MoTe_2$ bilayer were optimized with a force tolerance of 0.01 eV/Å followed by a subsequent optimization of atomic positions alone with a force tolerance of 0.001 eV/ Å. Periodic images were separated by ~14 Å vacuum normal to the layers to prevent spurious interactions. As standard DFT functionals are known to fail in describing interlayer van der Waals bonding correctly, we used the non-local optB86b van der Waals functional[4,5], which has been shown to reproduce the equilibrium geometry of $MoTe_2$ accurately[6]. Subsequent to structural optimization of the bilayer primitive cell, phonon dispersions were obtained within the harmonic approximation using Phonopy[7] and density functional perturbation theory (DFPT) in VASP. To ensure proper convergence of the force constants, a 4×4 supercell was employed in the DFPT calculations along with a tighter energy cutoff of 10^{-6} eV for electronic wavefunctions.

S3. Anti-Stokes Raman intensity and sample temperature calibration.

We use a suspended multilayer graphene (~10nm thick) to calibrate our optical system. In the photon range we are working with multilayer graphene has a flat optical response and we assume that its *R* in Equation (2) can be approximated by unity. At a given laser power we determine the temperature of the sample from Stokes and anti-Stokes Raman scattering of the G band, following Ref.[8]. Knowing the temperature, we then apply Equation (1) in the main text to compare the expected and measured Stokes and anti-Stokes Raman intensity; a typical spectra of the shear mode of the suspended graphene sample is shown in Fig.S1(a). The same sample is used to calibrate all three different laser excitations. At 532nm and 514nm the calibration values do not deviate significantly from 1 (1.00±0.04 and 0.99±0.04 respectively) since the Stokes and anti-Stokes breathing mode scattered photons have very similar energies (<0.3% difference) and the grating response is fairly flat; at 488nm the grating is more light wavelength sensitive and the calibration value is found to be 1.32±0.03. The uncertainties of calibration were propagated into the error bars in Fig. 4(b).

The Stokes anti-Stokes ratio depends on sample temperature since phonons are bosons as we

discussed in the main text. In general, absorption of laser light causes sample heating. As shown in Fig. S1 panels b & c for a 5L $MoTe_2$, the IMC and OMC peaks soften and broaden with laser heating for powers above 1 mW. To calibrate the temperature, we placed the same samples inside a cryostat with a built-in heater. The spectra were taken with incident laser power of 100 μW. Figure S1(d) shows the phonon energy of the IMC and OMC modes, which decrease linearly with heating. The slope is extracted by linear fitting as 0.01 cm^{-1}/K and 0.013 cm^{-1}/K with 3% uncertainty for IMC and OMC modes, respectively. For Raman data in Figures 3&4 the $MoTe_2$ samples are excited with a laser power of about 0.1mW. Comparing data in panel c with panel d, the laser heating effect at such power levels is very limited. We expect that for the power used in our experiment, the sample temperature is at most 20 K above room temperature. This defines the gray band in Fig.4(b) of the main text.

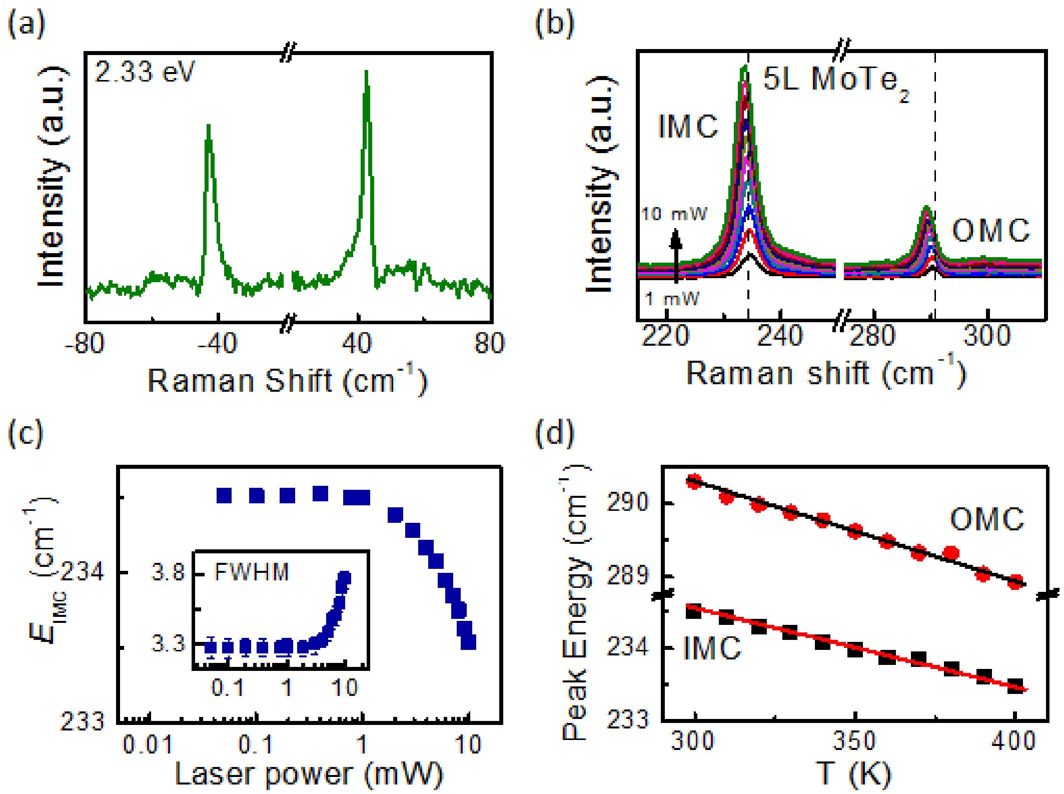

Figure S2. (a) Stokes and anti-Stokes Raman shear modes of suspended multilayer graphene. (b) Power dependent spectra of 5L $MoTe_2$. (c) Extracted peak energy and FWHM of IMC mode of 5L $MoTe_2$. (d) The temperature-dependent peak energy of the IMC and OMC modes.

References:

1. Kresse, G. & Furthmüller, J. Efficiency of ab-initio total energy calculations for metals and semiconductors using a plane-wave basis set. *Comput. Mater. Sci.* **6,** 15–50 (1996).

2. Blöchl, P. E. Projector augmented-wave method. *Phys. Rev. B* **50,** 17953–17979 (1994).

3. Kresse, G. From ultrasoft pseudopotentials to the projector augmented-wave method. *Phys. Rev. B* **59,** 1758–1775 (1999).

4. Klimeš, J., Bowler, D. R. & Michaelides, A. Van der Waals density functionals applied to solids. *Phys. Rev. B* **83,** 195131 (2011).

5. Dion, M., Rydberg, H., Schröder, E., Langreth, D. C. & Lundqvist, B. I. Van der Waals Density Functional for General Geometries. *Phys. Rev. Lett.* **92,** 246401 (2004).

6. Björkman, T. Testing several recent van der Waals density functionals for layered structures. *J. Chem. Phys.* **141,** 074708 (2014).

7. Togo, A. & Tanaka, I. First principles phonon calculations in materials science. *Scr. Mater.* **108,** 1–5 (2015).

8. Berciaud, S. *et al.* Electron and optical phonon temperatures in electrically biased graphene. *Phys. Rev. Lett.* **104,** 227401 (2010).
